# Time to Death and Its Predictors among Neonates Hospitalized with Sepsis in Eastern Ethiopia

**DOI:** 10.1155/2024/2594271

**Published:** 2024-04-05

**Authors:** Usmael Jibro, Assefa Desalew, Galana Mamo Ayana, Abera Kenay Tura

**Affiliations:** ^1^School of Nursing and Midwifery, College of Health and Medical Sciences, Haramaya University, Harar, Ethiopia; ^2^Department of Epidemiology and Biostatistics, School of Public Health, College of Health and Medical Sciences, Haramaya University, Harar, Ethiopia; ^3^Department of Obstetrics and Gynecology, University Medical Centre Groningen, University of Groningen, Groningen, Netherlands

## Abstract

**Background:**

Although neonatal sepsis is a major public health problem contributing to 30-50% of neonatal deaths in low- and middle-income countries, data on predictors of time to death are limited in Eastern Ethiopia. This study is aimed at determining predictors of time to death among neonates with sepsis admitted in public hospitals in Eastern Ethiopia.

**Methods:**

An institutional-based retrospective cohort study was conducted among 415 neonates admitted to referral hospitals in Eastern Ethiopia with sepsis from January 1, 2021, to December 31, 2021. Data were collected from medical records by using structured checklist and entered using EpiData 3.1 and analyzed using Stata 17. The Kaplan-Meier curves and log-rank tests were used to describe survival experience among different categories. The proportional hazard assumption and goodness of fit for the Cox regression model were checked. The Cox regression model was used to identify the significant predictors. Hazard ratios (HRs) with 95% confidence intervals (CI) were calculated. Finally, statistical significance was set at a *p* value < 0.05 in the Cox regression analysis.

**Results:**

Of the 415 neonates with neonatal sepsis, 71 (17.1%) (95% CI: 13.60-21.08) died at discharge, with a median time to death of 14 days. The overall incidence rate of mortality was 36.5 per 1000 neonate days. Low birthweight (AHR = 2.50; 95% CI: 1.15-5.44), maternal age ≥ 35 years (AHR = 3.17; 95% CI: 1.11, 9.04), low fifth-minute Apgar score (AHR: 2.32; 95% CI: 1.30-4.14), and late initiation of breastfeeding (AHR = 4.82; 95% CI: 1.40-16.65) were independent predictors of mortality among neonates with sepsis.

**Conclusions:**

Almost one in five neonates with sepsis died at discharge. Low birthweight, maternal age ≥ 35 years, low fifth-minute Apgar score, and late initiation of breastfeeding were predictors of mortality.

## 1. Background

Globally, 2.4 million neonates die during first month of life every year, mostly from preventable causes [1]. Of these, approximately 50% dies on the first day and 75% dies within the first week of life [2, 3]. One of the leading causes of neonatal death is neonatal sepsis, contributing to 15% of neonatal mortality worldwide [4] and 30-50% in low- and middle-income countries (LMICs) [5, 6]. Neonatal sepsis, defined as a positive blood culture within the first 28 days of birth, is a systemic inflammatory response syndrome associated with an infection, either proven or suspected bacteremia [7].

According to the global burden of disease estimates, 3 million neonatal sepsis cases and 500,000 sepsis-related deaths were reported worldwide in 2020 [8]. Most of these cases occur in LMICs [6]. Ethiopia has a high burden of neonatal sepsis, with approximately 37% of all neonatal mortality attributed to neonatal sepsis [9]. Moreover, survivors of neonatal sepsis often suffer from short- and long-term neurologic and developmental vulnerabilities that affect their overall growth and development, performance, altered quality of life, and increased healthcare costs [10, 11]. Despite a declining neonatal mortality rate globally, neonatal mortality is still high in LMICs [12].

Different studies showed that being low birthweight, preterm, hypothermic, asphyxiated at birth, having fever, and other comorbidities on admission are significant predictors of mortality among neonates with sepsis [13–16]. Although several studies on the prevalence, risk factors, and mortality among neonates with sepsis exist, including in Ethiopia, there is limited evidence regarding time to death and its predictors among neonates with neonatal sepsis in Ethiopia. Knowledge of time to death and its predictors is essential for designing tailored interventions to prevent high sepsis-caused neonatal mortality. Therefore, this study assessed time to death and predictors among neonates admitted to neonatal intensive care units (NICU) in two major public hospitals in Eastern Ethiopia.

## 2. Methods and Materials

### 2.1. Study Settings, Design, and Population

An institutional-based retrospective cohort study was conducted with neonates admitted with sepsis in the two major public referral hospitals in Eastern Ethiopia: Hiwot Fana Comprehensive Specialized University Hospital and Dil Chora Referral Hospital. Hiwot Fana Comprehensive Specialized University Hospital is a teaching referral hospital of Haramaya University, located in Harar Town, serving for more than six million people in Eastern Ethiopia. Approximately, 1700 neonates are admitted to the NICU annually. Dil Chora Referral Hospital is a governmental referral hospital located in Dire Dawa administration, approximately 60 kilometers away from Harar. All neonates who were admitted to the NICU with neonatal sepsis from January 1, 2021, to December 31, 2021, in both hospitals were included, whereas neonates with incomplete medical records especially outcome at discharge was missing were excluded from the study. The study was conducted from March 20 to April 19, 2022.

The sample size was calculated using Epi Info version 7.2 using double population proportion in Stata statistical program considering the following assumptions: *p* (proportion of mortality among neonates admitted to NICU with sepsis), 11.6% [15], 95% CI, a margin of error (5%), power of 80%, and proportion of exposed to unexposed with the outcome, *Zβ*, 1 : 1(*n* = 432). The sample size was proportionally allocated to the selected hospitals based on the number of NICU admissions. Using the medical registration number of all neonates admitted with neonatal sepsis during the study period (*n* = 3114) as the sampling frame, a simple random sampling technique was used to select final samples (*n* = 432).

### 2.2. Data Collection

Data were collected through a review of medical records using a pretested structured validated data abstraction checklist [17]. The checklist contains information on the sociodemographic conditions of the mother and neonate, obstetric factors, treatments received, and neonatal status at discharge or 28 days, whichever comes first. Neonatal sepsis was considered as defined by the attending clinicians. Trained nurses with experience in data collection collected the data under the supervision of principal investigators. Any infant born before 37 weeks of gestational age was considered preterm, and birthweight < 2500 g was the cutoff point for low birthweight. Two days of training was provided to the data collectors.

### 2.3. Data Processing and Analysis

After checking for completeness, the data were entered in EpiData 3.1 and analyzed using Stata 17. Continuous variables were described using mean (standard deviation) or median as appropriate, while frequencies and percentages were used for categorical variables. The incidence density rate (IDR) was calculated for the entire study period and reported per 1000 neonatal days. The Kaplan-Meier analysis was used to estimate the cumulative probability of failure, and log-rank test was used to compare the survival time differences for categorical variables. Before fitting the Cox regression model, the proportional hazard assumption was verified using the Schoenfeld residual test. The bivariate Cox regression model was fitted for each explanatory variable, and variables with *p* ≤ 0.25 were candidates for the multivariable Cox regression model. A hazard ratio along with its 95% confidence interval was used to measure the strength of the association. Finally, a *p* value < 0.05 in the adjusted Cox regression was considered statistically significant.

## 3. Results

### 3.1. Neonatal-Related Characteristics

Of the 432 neonate medical records reviewed, 415 (96%) which fulfilled the inclusion criteria were included in the final analysis: 8 were incomplete and 9 were not retrievable. The median age of the neonates at admission was 1 day with interquartile range of 2 days, ranging from 1 to 21 days. The mean weight of neonates at admission was 2586.6 (±666.4) gram, ranging from 1000 to 4500 gram, and the mean gestational age was 37.1 (±2.4) weeks ranging from 28 to 43 weeks. Two-thirds (66.5%) of the neonates had other comorbidities (Table 1).

### 3.2. Maternal Sociodemographic, Medical, and Obstetrics Characteristics

Two-thirds (65%) of the mothers were multigravida. The majority of the women had antenatal care (ANC) visits (97.8%), gave birth in health institutions (95.66%), and gave birth through spontaneous vaginal delivery (70.36%). Almost half of the women had spontaneous onset of labor (45%). The mean age of mothers was 26.4 (±4.3) years, ranging from 17 to 38 years with (90.4%) belonging to the age group of 20-35 years (Table 2).

### 3.3. Treatment-Related Characteristics

The majority of the sepsis was diagnosed using complete blood count (CBC) (398; 95.9%). The most identified organism during culture was E. coli (15, 71.4%). Most neonates (91.6%) were treated with a combination of ampicillin and gentamycin. There was significant difference regarding feeding methods and other treatments among the neonates who survived and died (Table 3).

### 3.4. Time to Death and Survival Status

**Figure 1:**
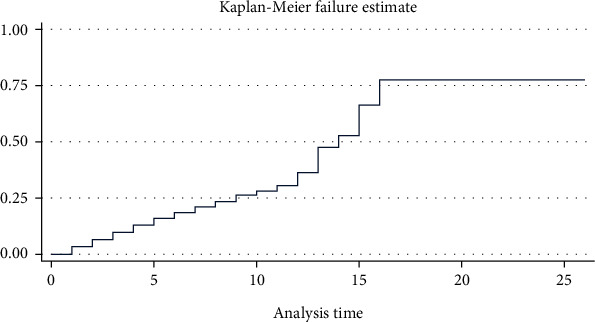
Overall Kaplan-Meir failure estimate of neonates admitted with neonatal sepsis in selected public referral hospitals in Eastern Ethiopia, 2022 (*n* = 415).

From the 415 neonates followed from 1 to 26 days, a total of 1942 neonatal days of observation was observed. At the end of follow-up period, 71 (17.1%) (95% CI: 13.6, 21.08) neonates had died. The overall median time to death was 14 days (95% CI: 13, 16) (Figure 1). The probability of death was higher during the first and second week. After the second week, the graph moved upward rapidly and became straight indicating a continued high probability of death for some days and remained constant with almost no deaths occurring for the remaining follow-up time. From all deaths, 14 (19.7%), 35 (49.3%), and 56 (78.9%) deaths occurred within the first 24 hours, three days, and seven days of admission, respectively.

The overall incidence rate of mortality was 36.5 per 1000 neonate days (95% CI: 28.97, 46.13). At the end of follow-up time, the cumulative failure probability was 77.51% (95% CI: 53.6, 94.5). Among censored neonates, 77.4%, 4.34%, and 1.2% were improved and discharged, against medical treatment, and referred to other hospitals, respectively.

**Figure 2:**
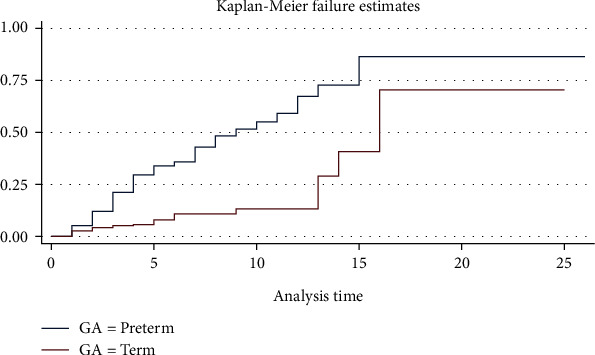
The Kaplan-Meier survival curves that compare the survival time of neonates with neonatal sepsis categories of gestational age of neonates admitted in selected public referral hospitals in Eastern Ethiopia, 2022 (*n* = 415).

**Figure 3:**
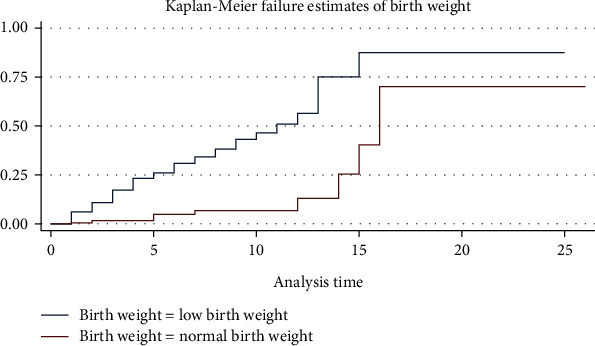
The Kaplan-Meier survival curves compare the survival time of neonates with neonatal sepsis categories of birthweight of neonates admitted in selected public referral hospitals in Eastern Ethiopia, 2022 (*n* = 415).

In the log-rank test, the survival pattern varied significantly among covariates. As such, gestational age (Figure 2), birthweight (Figure 3), first and five-minute Apgar score, resuscitation at birth, preterm labor, pregnancy-induced hypertension, initiation of breastfeeding, antenatal visit, and history of premature rupture of membranes had a significant effect on neonatal mortality among neonates with neonatal sepsis at *p* < 0.05 (Table 4).

The Cox proportional hazard assumption was checked by global test (Schoenfeld residual test). For each variable, the Cox proportional hazard assumption was done individually and simultaneously (globally). The test revealed that there were no time-varying covariates in the model because both the *p* values for each covariate individually and the total covariates jointly were greater than 0.05 (Table 5).

### 3.5. Testing Overall Fitness of the Model

**Figure 4:**
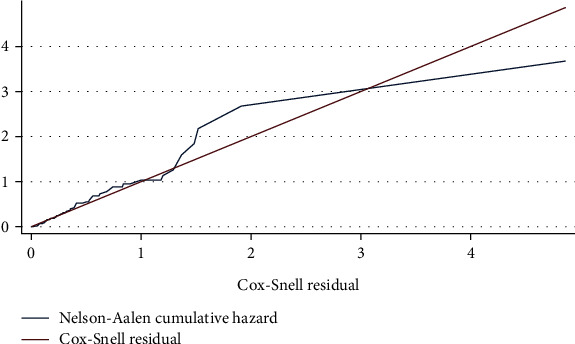
The Cox-Snell residual Nelson-Aalen graph showing model goodness of fit for neonates admitted with neonatal sepsis in referral hospitals in Eastern Ethiopia, 2022 (*n* = 415).

The Cox-Snell residual test was used to evaluate the model's goodness of fit. The residuals had a standard censored exponential distribution with a hazard ratio. The 45-degree line is closely followed by the jagged line with the reference line (Cox-Snell residual line). Therefore, the overall Cox regression model fits the data (Figure 4).

### 3.6. Predictors of Time to Death

In the multivariable Cox regression model, initiation of breastfeeding, fifth-minute Apgar score, maternal age ≥ 35 years, and birthweight were independent predictors of mortality. Neonates with low birthweight (<2500 grams) had a 2.62 (AHR = 2.62; 95% CI: 1.20-5.74) times higher risk of death compared to neonates with normal birthweight. Neonates who were born from mothers > 35 years had 3.17 times higher risk of mortality as compared to those born from mothers < 35 years of age (AHR = 3.17; 95% CI: 1.11, 9.04). Similarly, neonates who were not breastfed within one hour had a 4.42 (AHR = 4.42; 95% CI: 1.32-14.82) times risk of death than their counterparts. Moreover, the hazard of death among newborns with a fifth-minute Apgar score < 7 was 2.32 times (AHR = 2.53; 95% CI: 1.41–4.53) higher than neonates with fifth-minute Apgar scores 7-10 (Table 6).

## 4. Discussion

This study was conducted to determine the time to death and its predictors among neonates with neonatal sepsis admitted to NICU in public referral hospitals in Eastern Ethiopia. We found that one in six neonates admitted with neonatal sepsis died at discharge corresponding with an incidence rate of 36.5 (95% CI: 28.97, 46.13) per 1000 neonate days. The overall median time to death was 14 days. Predictors of mortality among neonates with neonatal sepsis were low birthweight, maternal age ≥ 35 years, low fifth-minute Apgar score, and late initiation of breastfeeding.

The overall mortality rate (36.5 per 1000 neonate days) was similar to that reported in a study conducted at the University of Gondar Comprehensive Specialized Hospital in Ethiopia (34.69) [18]. However, it is higher than the findings from other findings in Ethiopia: Arba Minch (14.57) [15], Bahir Dar (20.5) [16], and Wollega (6.81) [19]. This might be related to variations in the follow-up period. For instance, in our study, the neonates were followed up for the entire neonatal period, but the other maximum follow-up period was 14 days.

The overall median time to death of neonates (14 days) in our study is higher than those reported in Brazil [14] and Wollega, Ethiopia [19]. This might be explained by the discrepancy in the follow-up time or the characteristics of the study population. For instance, the study from Brazil included only neonates with positive blood culture. Consistent with the previous findings, most neonatal deaths occurred within the first week of admission [15, 16, 19]. Thus, improving the survival of neonates requires attention during the first few critical days.

Not surprisingly, low birthweight neonates have a higher risk of mortality than term and normal birthweight neonates. Similar findings were reported in a systematic review and meta-analysis of mortality from neonatal sepsis [6], and other studies conducted in Brazil [14], Iraq [20], Central India [21], Pakistan [22], and Ethiopia [15, 23, 24]. This is because low birthweight may relate with prematurity; the immune system of preterm neonates is not well developed. In addition, preterm and low birthweight neonates are susceptible to hypothermia and respiratory distress syndrome due to immature thermoregulatory centers and lung immaturity [21]. Thus, helping the baby breathe, kangaroo mother care, and keeping the baby warm are beneficial for increasing the survival of premature newborns [25].

Moreover, late initiation of exclusive breastfeeding was a significant predictor of mortality. This is in line with studies conducted in India [26], Zimbabwe [27], a systematic review in LMICs [28], and Ethiopia [15, 16]. Early initiation of exclusive breastfeeding, which provides adequate nutrition and prevents hypothermia and hypoglycemia, is the major cause of neonatal mortality, especially among preterm and low birthweight [29]. In addition, colostrum prevents the penetration of pathogenic microorganisms into the gut, resulting in necrotizing enterocolitis. The high levels of immunoglobulins and lymphocytes found in the colostrum stimulate the immune response of newborns during early infection [30]. As such, early initiation of breastfeeding is beneficial for improving the survival of neonates with sepsis.

The study finding showed that advanced maternal age (≥35 years) was a significant predictor of mortality. This is in line with the studies conducted in Uganda [31] and Ethiopia in Amhara region referral hospitals [32], in Gamo Gofa [33], and in Eastern Ethiopia [34]. This might be the fact that advanced maternal age increases the risk of gestational diabetes mellitus which predisposes the neonates to assisted ventilation and NICU admission [35].

Furthermore, we found that neonates with low fifth-minute Apgar scores had a higher risk of mortality. This is in line with studies conducted in Scotland [36], Cameron [37], and Ethiopia [38]. The fifth-minute Apgar score shows the neonate's capacity to survive and thrive, and low Apgar scores at 5 minutes indicate an increased risk of cerebral palsy that occurred after severe birth asphyxia [39, 40]. This might be due to a delay in the identification of newborn complications, and their management as the fifth-minute Apgar score indicates the success of neonatal resuscitation.

## 5. Limitations of the Study

Our study has some limitations to be considered during interpretation. *First*, some potential sociodemographic predictors are not routinely documented in medical records. *Second*, the follow-up was limited to discharge or 28 days, whichever comes first. Thus, deaths among neonates discharged before 28 days might be missed.

## 6. Conclusions

One in six neonates who were admitted with neonatal sepsis to neonatal intensive care units in referral hospitals in Eastern Ethiopia died at discharge. The overall incidence rate of mortality was relatively high. Low birthweight, maternal age ≥ 35 years, fifth-minute Apgar score < 7, and late initiation of breastfeeding were statistically significant predictors of mortality. Strengthening immediate newborn care, including resuscitation, continuous care for preterm, low birthweight, and timely initiation of breastfeeding are essential for preventing the high mortality among newborns with neonatal sepsis. Further studies on the appropriateness of care for neonates with neonatal sepsis are warranted for improving the quality of care and thereby survival of neonates.

## Figures and Tables

**Table 1:** Neonatal-related characteristics of neonates admitted with neonatal sepsis in selected public referral hospitals in Eastern Ethiopia, 2022 (*n* = 415).

Variables	Category	Status of the neonate	
		Death	Censored
Neonatal age (days)	<7	68 (17.85%)	313 (82.15%)
	7-28	3 (8.82%)	31 (91.18%)
Neonatal sex	Male	41 (16.6%)	206 (83.4%)
	Female	30 (17.86%)	138 (82.14%)
Residence	Urban	38 (18.54%)	167 (81.46%)
	Rural	33 (15.71)	177 (84.29%)
Gestational age (*n* = 391)	Preterm	45 (38.8%)	71 (61.2%)
	Term	22 (8%)	253 (92%)
Admission weight in grams	<2500	60 (28.4%)	151 (71.6%)
	≥2500	11 (5.4%)	193 (94.6%)
First-minute Apgar score (*n* = 397)	<7	55 (23.1)	183 (76.9%)
	≥7	12 (7.6%)	147 (92.5)
Fifth-minute Apgar score (*n* = 397)	<7	43 (38.4%)	69 (61.6%)
	≥7	24 (8.4%)	261 (91.6%)
Cry immediately at birth	Yes	10 (5%)	183 (95%)
	No	61 (27.5%)	161 (72.5%)
Resuscitated at birth	Yes	61 (26.8%)	167 (72.5%)
	No	10 (5.4%)	177 (94.6%)
EBF initiated within one hour (*n* = 205)	Yes	3 (3.7%)	78 (96.3%)
	No	11 (8.9%)	113 (91.1%)
Comorbidity	Yes	64 (23.2%)	212 (76.8%)
	No	7 (5%)	132 (95%)

**Table 2:** Maternal sociodemographic, medical, and obstetric-related characteristics of neonates admitted with neonatal sepsis in selected public referral hospitals in Eastern Ethiopia, 2022 (*n* = 415).

Variables	Category	Status of the neonate	
		Death	Censored
Maternal age in years	<20	6 (21.43%)	22 (78.57)
	20-34	61 (16.27%)	314 (83.73)
	≥35	4 (33.3%)	8 (66.6%)

Parity	Primipara (1)	25 (18.2%)	112 (81.8%)
	Multipara (1-5)	42 (16.3%)	215 (83.7%)
	Grand multipara (≥5)	4 (19%)	17 (81%)

Number of ANC visits (*n* = 406)	1-3	43 (21.9%)	153 (78.1%)
	≥4	27 (12.9%)	183 (87.1%)

Onset of labor	Spontaneous	26 (12.5%)	182 (87.5%)
	Induced	22 (20%)	88 (80%)
	CS before labor	23 (23.7%)	74 (76.3%)

Place of delivery	Health facility	69 (17.4%)	328 (82.6%)
	Home	2 (11.1%)	16 (88.9%)

Mode of delivery	SVD	41 (14%)	251 (86%)
	CS	28 (24.6%)	86 (75.4%)
	Instrumental delivery	2 (22.2%)	7 (77.8%)

History of PROM	Yes	19 (36.5%)	33 (63.5%)
	No	52 (14.3)	311 (85.7%)

Duration of PROM (*n* = 52)	<18 hours	13 (46.4%)	15 (53.6%)
	≥18 hours	6 (25%)	18 (75%)

PPH	Yes	4 (13.8%)	25 (86.2%)
	No	67 (17%)	319 (83%)

APH	Yes	8 (14.8%)	46 (85.2%)
	No	63 (17.5%)	298 (82.5%0

PIH	Yes	32 (28.8%)	79 (71.2%)
	No	39 (12.8%)	265 (87.2%)

Preterm labor	Yes	37 (39%)	58 (61%)
	No	34 (10.6%)	286 (89.4%)

Multiple (twin)	Yes	5 (33.3&)	10 (66.7%)
	No	66 (16.5%)	334 (83.5%0

Previous CS	Yes	7 (16.7%)	35 (83.3%)
	No	64 (17%)	309 (83%)

Abnormal presentation	Yes	5 (18.5%)	22 (81.5%)
	No	66 (17%)	322 (83%)

STIs	Yes	5 (27.8%)	13 (72.2%)
	No	66 (16.6%)	331 (83.4%)

Chronic medical illness of the mother	Yes	24 (19.5%)	99 (80.5%)
	No	47 (16%)	245 (84%)

PROM: premature rupture of membrane; PPH: postpartum hemorrhage; APH: antepartum hemorrhage; PIH: pregnancy-induced hypertension; STI: sexually transmitted infection; CS: cesarean section; SVD: spontaneous vaginal delivery; ANC: antenatal care.

**Table 3:** Treatment-related characteristics of neonates admitted with neonatal sepsis in selected public referral hospitals in Eastern Ethiopia, 2022 (*n* = 415).

Variables	Category	Status of the neonate	
		Death	Censored
CBC	Yes	66 (16.6%)	332 (83.4%)
	No	5 (29.4%)	12 (70.6%)

Culture	Yes	1 (5%)	20 (95%)
	No	70 (18%)	324 (82%)

Identified causative agent (*n* = 21)	E.coli	1 (7%)	14 (93%)
	Klebsiella	0	6 (100%)

Type of antibiotics given	Ampicillin+gentamicin	68 (18%)	312 (82%)
	Gentamicin+ceftriaxone	3 (22%)	10 (78%)
	Ampicillin+gentamicin+vancomycin	3 (37.5%)	5 (62.5%)

Duration of antibiotics	<7 days	54 (17%)	269 (83%)
	≥7 days	17 (18%)	75 (82%)

Feeding method	Breastfeeding	4 (7.1%)	52 (92.9%)
	Express feeding	16 (10%)	144 (90%)
	Formula feeding	51 (25.6%)	148 (74.4%)

Other treatments/procedures	Blood transfusion	5 (45)	6 (55%)
	Incubator	25 (38%)	41 (62%)
	Glucose	53 (22.6%)	181 (77.4%)
	Resuscitation	41 (21%)	152 (79%)
	Phototherapy	26 (40.6%)	38 (59.4%)

CBC: complete blood count.

**Table 4:** Log-rank test of different categorical predictors of neonates admitted with neonatal sepsis in selected public referral hospitals in Eastern Ethiopia, 2022 (*n* = 415).

Variable	Category	*X* ^2^	*p* value
Gestational age	Preterm	63.09	0.00001
	Term		
Neonatal sex	Male	0.10	0.7536
	Female		
Birthweight	<2500	32.55	0.00001
	≥2500		
Five-minute Apgar score	<7	63.14	0.00001
	≥7		
Maternal residence	Urban	0.28	0.5959
	Rural		
Cry at birth	No	39.19	0.00001
	Yes		
First minute Apgar score	<7	42.82	0.0001
	≥7		
Preterm labor	Yes	32.92	0.0001
	No		
Place of delivery	Health facility	0.56	0.4533
	Home		
PIH	Yes	10.97	0.0009
	No		
EBF initiated within one hour	Yes	10.23	0.0014
	No		
ANC visit	1-3 visits	5.56	0.0183
	≥4 visits		
History of PROM	Yes	19.32	0.00001
	No		

**Table 5:** Test of the Cox proportional hazard assumption among neonates admitted with neonatal sepsis at public referral hospitals, Eastern Ethiopia, 2022 (*n* = 415).

Variable	Rho	Chi2	df	Prob>chi2
Neonatal age	0.06175	0.24	1	0.6214
Maternal age	0.01829	0.03	1	0.8715
Neonatal sex	-0.03345	0.10	1	0.7513
Gestational age	-0.03506	0.11	1	0.7429
Weight at admission	-0.03863	0.12	1	0.7245
First-minute Apgar score	0.05809	0.24	1	0.6267
Five-minute Apgar score	-0.06266	0.29	1	0.5887
ANC visit	0.04465	0.19	1	0.6650
History of PROM	0.02611	0.08	1	0.7756
PPH	0.02327	0.04	1	0.8405
APH	-0.02786	0.06	1	0.7995
PIH	-0.06404	0.48	1	0.4903
Preterm labor	0.12592	1.53	1	0.2156
Previous cesarean section scar	0.06500	0.32	1	0.5733
STIs	-0.03707	0.12	1	0.7317
Mode of delivery	-0.03469	0.11	1	0.7390
Onset of labor	-0.01360	0.02	1	0.8818
The medical condition of the mother	0.04566	0.19	1	0.6631
Gravidity	0.10207	0.87	1	0.3506
Parity	-0.04426	0.16	1	0.6911
Comorbidity	-0.00437	0.00	1	0.9667
Resuscitated at birth	-0.04991	0.03	1	0.8730
EBF initiated within one hour	0.02142	0.04	1	0.8482
Cry at birth	-0.11330	0.09	1	0.7595
Global test		6.78	24	0.9998

PPH: postpartum hemorrhage; APH: antepartum hemorrhage; PIH: pregnancy-induced hypertension; STI: sexually transmitted infection; Apgar: appearance, pulse, grimace activity, and respiration.

**Table 6:** The Cox proportional hazard regression for identifying predictors of mortality among neonates with sepsis admitted into referral hospitals in Eastern Ethiopia, 2022 (*n* = 415).

Variable	Category	CHR (95% CI)	AHR (95% CI)	*p* value
Maternal age	≥35 years	3.51 (1.27–9.74)	3.17 (1.11-9.04)^∗∗^	0.031
	<20 years	1.35 (0.58–3.12)	0.69 (0.28-1.70)	0.424
	20-35 years	1	1	

Birthweight	<2500 g	6.20 (3.25–11.82)	2.62 (1.20–5.74)^∗∗^	0.017
	≥2500 g	1	1	

First-minute Apgar score	<7	4.39 (2.67–7.15)	0.31 (0.04–2.48)	0.271
	≥7	1	1	

Five-minute Apgar score	<7	5.91 (3.61–9.68)	2.53 (1.41–4.53)^∗∗^	0.002
	≥7	1	1	

ANC visit	1-3 visits	1.75 (1.08–2.83)	1.23 (0.73–2.07)	0.444
	≥4 visits	1	1	

History of PROM	Yes	3.07 (1.80-5.24)	1.38 (0.73-2.59)	0.319
	No	1	1	

PIH	Yes	1.93 (1.20–3.09)	0.97 (0.53–1.81)	0.944
	No	1	1	

Comorbidity	Yes	4.89 (2.24-10.67)	1.77 (0.74-4.20)	0.175
	No	1	1	

Initiation of breastfeeding one hour	No	5.27 (1.66-16.78)	4.42 (1.32-14.82)^∗∗^	0.016
	Yes	1	1	

CHR: crude hazard ratio; PROM: premature rupture of membrane; PPH: postpartum hemorrhage; APH: antepartum hemorrhage; PIH: pregnancy-induced hypertension; STI: sexually transmitted infection; CS: cesarean section; SVD: spontaneous vaginal delivery; ANC: antenatal care. ^∗∗^ shows significance at a *p*-value less than 0.05, AHR: adjusted hazard ratio.

## Data Availability

Upon reasonable request, the corresponding author will provide all study data.

## Abbreviations

AHR: Adjusted hazard ratio
ANC: Antenatal care
Apgar: Appearance, pulse, grimace activity, and respiration
CI: Confidence interval
EOS: Early-onset neonatal sepsis
LOS: Late-onset sepsis
LMICs: Low- and middle-income countries
NICU: Neonatal intensive care units.
